# Technologies for Managing the Health of Older Adults with Multiple Chronic Conditions: A Systematic Literature Review

**DOI:** 10.3390/healthcare11212897

**Published:** 2023-11-03

**Authors:** Gabriela Cajamarca, Valentina Proust, Valeria Herskovic, Rodrigo F. Cádiz, Nervo Verdezoto, Francisco J. Fernández

**Affiliations:** 1 School of Mathematical and Computational Sciences, Yachay Tech University, San Miguel de Urcuquí 100119, Ecuador; mcajamarca@yachaytech.edu.ec; 2 Annenberg School for Communication, University of Pennsylvania, Philadelphia, PA 19104, USA; 3 Department of Computer Science, School of Engineering, Pontificia Universidad Católica de Chile, Santiago 7820436, Chile; 4 Department of Electrical Engineering, School of Engineering, and Music Institute, Faculty of Arts, Pontificia Universidad Católica de Chile, Santiago 7820436, Chile; rcadiz@uc.cl; 5 School of Computer Science and Informatics, Cardiff University, Cardiff CF24 4AG, UK; VerdezotoDiasN@cardiff.ac.uk; 6 Faculty of Communication, Pontificia Universidad Católica de Chile, Santiago 8320000, Chile; ffernandez@uc.cl

**Keywords:** aging, older adults, chronic disease, multimorbidity, technologies

## Abstract

Multimorbidity is defined as the presence of two or more chronic medical conditions in a person, whether physical, mental or long-term infectious diseases. This is especially common in older populations, affecting their quality of life and emotionally impacting their caregivers and family. Technology can allow for monitoring, managing, and motivating older adults in their self-care, as well as supporting their caregivers. However, when several conditions are present at once, it may be necessary to manage several types of technologies, or for technology to manage the interaction between conditions. This work aims to understand and describe the technologies that are used to support the management of multimorbidity for older adults. We conducted a systematic review of ten years of scientific literature from four online databases. We reviewed a corpus of 681 research papers, finally including 25 in our review. The technologies used most frequently by older adults with multimorbidity are mobile applications and websites, and they are mostly focused on communication and connectivity. We then propose opportunities for future research on addressing the challenges in the management of several simultaneous health conditions, potentially creating a better approach than managing each condition as if it were independent.

## 1. Introduction

The increase in the number of older adults, as well as the percentage of the population they represent, is a societal transformation that has created challenges for countries in various areas, particularly in the provision of health services [1]. Older adults with multimorbidity—the coexistence of two or more chronic health conditions [2]—represent a significant portion of the primary health care population [3,4]. Having multiple chronic conditions is associated with individual adverse outcomes, such as decreased quality of life [5], reduced physical functioning [5,6], and increased re-hospitalization and mortality rates [7]. Multimorbidity affects not only the person, but also their caregivers and family environment [8,9]. Furthermore, multimorbidity presents a challenge for healthcare professionals’ decision-making practices—e.g., a recent review explored technological support for physicians diagnosing patients with multimorbidity, finding computer-based simulation of clinical cases can be efficient in developing clinical reasoning for multimorbidity [10].

Managing multiple health conditions is challenging for patients, e.g., in the understanding and management of their diseases, e.g., attending multiple appointments and managing therapies or medications [11,12]. Patients must handle a large amount of information about each condition and represent their own interests [13], as well as be aware of any potential interactions between their conditions or between their medications. Furthermore, patients with multimorbidity may feel anxiety and distress towards their conditions, and sometimes cope through avoidance [14] so there may be challenging barriers to provide treatment as well as to implement interventions to support them.

Health technology encompasses a wide range of technology use for medicine, including e.g., drug development, telemedicine, and computer vision for medical images. In this paper, we focus on interactive health technologies geared towards an end user, i.e., technologies that enable a user (patient, caregiver, or healthcare professional) to interact with digital health information, supporting information exchange and self-care [15]. These technologies can be used by older adults to facilitate monitoring and management of their health conditions [16]. For example, mobile health technologies can provide patients with personalized and engaging solutions, at a low cost [17].

Interactive health technologies should be designed considering their users’ specific needs and constraints. User-centered design is an approach that involves users in design and development, with the aim of ensuring that their needs regarding technologies are met [15]. However, little emphasis has been placed on technology solutions aimed at older adults [18], especially those with multiple chronic diseases, as most digital solutions aimed at older adults support single-disease management, e.g., diabetes [19] or cardiovascular disease [20]. Recently, one study on the use of health technologies by adults with multimorbidity found that they use these technologies mainly to make medical decisions and communicate with health providers—however, older adults were found to be less likely to use them [21].

Although other systematic literature reviews have been carried out to account for the state of use of interactive health technologies by older adults, these do not focus on multimorbidity, concentrating instead on particular chronic diseases or conditions (e.g., [22,23,24]). However, managing multiple chronic conditions is not the same as managing them individually, as there may be complex inter-relations between the conditions, as well as within their management. We aim to study which interactive health technologies for older adults with multimorbidity have been proposed, to extract insights and lessons that may help guide development of these types of technologies in the future. For this purpose, this work presents a systematic review of the literature regarding technological solutions to address the needs of older adults with multiple chronic conditions. We aim to identify the type, support, and evaluation of technological tools that are used or proposed to support the health management of older adults with multimorbidity. Therefore, our research questions (RQ) are the following: **RQ1:** Which types of technologies have been developed to support the health management of older adults with multiple health conditions? **RQ2:** What are the expected health-related outcomes of technological interventions for older adults with multimorbidity? and **RQ3:** How are technologies for older adults with multiple health conditions evaluated?

This document is organized as follows. In Section 2, we describe the method used to locate and select scientific papers for our review. In Section 3, we present the results as answers to our research questions. Then, in Section 4, we discuss our results; we present the conclusions in Section 5.

## 2. Systematic Literature Review Methodology

This work is a systematic literature review (SLR) of studies focused on technology for the health management of older adults with multiple chronic conditions. The SLR method involves collecting articles to organize, analyze, and identify the essential gaps to be addressed in future work [25]. Our work follows the systematic review steps of (1) question formulation; (2) locating studies; (3) study selection and evaluation; (4) analysis and synthesis; and (5) reporting and using results [26]. Each of these steps are described below. This work also follows PRISMA (Preferred Reporting Items for Systematic Reviews and Meta-Analyses) guidelines [27] to ensure the review is replicable and systematically sound.

### 2.1. Question Formulation

We first defined the general concepts to formulate the research questions (see Table 1) by using the PICOC method (Population, Intervention, Comparison, Results, Context) [28] The population of interest in this study is older adults. According to the United Nations, older adults are those who are over 60 years old, although other definitions consider older adults to be over 65 [29,30]. The context is multimorbidity, i.e., those who have multiple health conditions. We are interested in studying interventions for the management of multimorbidity using technology, and we are interested in how the effects on health of these technologies are assessed. We did not focus on comparing technologies nor outcomes.

### 2.2. Locating Studies

**Figure 1:**
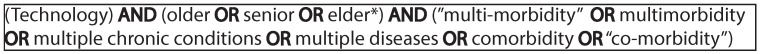
Search String.

For the search, we considered studies published in ten years (from 2009 to 2019) in the following databases: ACM Digital Library, IEEE Xplore, ScienceDirect, and Pubmed. We believe that combining these four sources provides a comprehensive representation of research on technologies for older adults with multiple chronic conditions, both from a computer science and a health informatics perspective. The terms and synonyms used as the search string (stemming from the PICOC terms in Table 1) are presented in Figure 1. The Boolean operator “OR” is used to select alternative terms and synonyms and the Boolean operator “AND” is used to add terms to the string. We use quotation marks (“ ”) to search for an exact match of compound words. The asterisk operator (*) indicates that there may be more letters after the root word. The search strings were applied to titles and abstracts.

The initial search yielded 681 papers, out of which 161 were duplicate entries, resulting in 520 potential papers. After this step, we applied inclusion and exclusion criteria to further examine which articles were relevant for our search. This step is explained in the next section.

### 2.3. Study Selection and Evaluation

After obtaining the studies, as part of our study selection and evaluation, inclusion and exclusion criteria were applied to filter out research that was not relevant for our search. For this purpose, we included articles that fulfilled the following eligibility criteria: (1) published in peer-reviewed scientific journals or conferences, (2) written in English, (3) published between 2009 and 2019, (4) presenting technology (implementations, prototypes, or design concepts) specifically proposed for older adults with multiple chronic conditions. Exclusion criteria were as follows: (1) research about technologies aimed at a single chronic condition, (2) publications written in languages other than English, (3) abstracts, summaries, invited plenary sessions, letters to the editor, or reviews, or (4) if the article was not available for download. We did not assess the quality of the selected studies, opting to include all the studies that complied with our inclusion and exclusion criteria.

### 2.4. Analysis and Synthesis

The data extraction procedure we used has four phases of selection and a final phase of data aggregation. First, we performed an initial search and filtering, for which we used the search strings defined previously, stored the resulting documents in a repository, and removed the duplicates. Second, two authors (GC and VP) read the titles and abstracts of 10 randomly selected papers and applied the inclusion and exclusion criteria. A meeting resolved disagreements about the inclusion and exclusion criteria. After this calibration step, the same authors (GC and VP) read all the titles and abstracts of the selected papers, indicating which studies met these criteria. Articles with two inclusion or exclusion votes were automatically included or excluded. Articles that had one acceptance and one rejection vote were reviewed and resolved by a third reviewer (VH). Third, two authors (GC and VP) read the full text of each of the selected articles. Relevant information was extracted into a Google spreadsheet during this phase. Finally, the extracted data were compared, and disagreements were discussed in an online meeting.

### 2.5. Reporting and Using Results

The next section presents our results. We answer our three research questions in Section 3.1, Section 3.2 and Section 3.3. Section 4 discusses our findings, limitations, and insights, e.g., regarding opportunities to improve technological support for older adults with multimorbidity. In this way, we provide ways to use our results in future work.

## 3. Results

**Figure 2:**
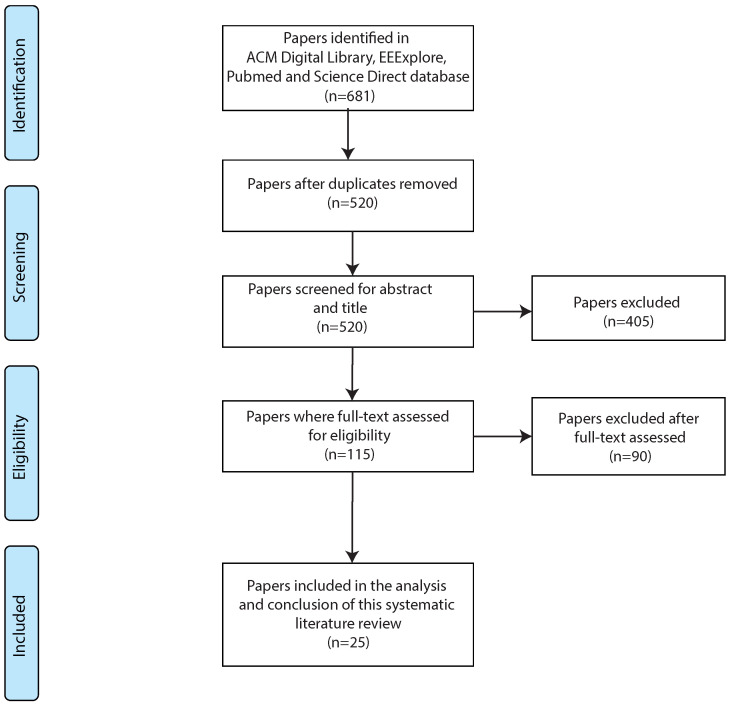
Flow chart showing study selection.

This section presents the results obtained from the systematic literature review. The selection and filtering process is presented in Figure 2. As shown, our initial search yielded a total of 681 items, which became 520 after the duplicates were removed. After reviewing title and abstract to eliminate studies that did not meet the inclusion/exclusion criteria, 405 articles that did not meet the criteria were removed from the corpus. Subsequently, 115 articles were assessed for eligibility through full-text analysis, and finally, 25 publications met the inclusion criteria.

**Figure 3:**
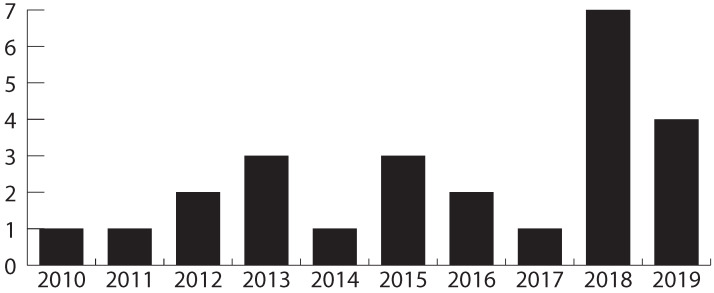
Frequency of publications per year.

The selected studies were published between 2009 and 2019 (per our search criteria), with 44% of them being published after 2017. Figure 3 shows the distribution of articles published over time. Twelve of the selected articles (12/25, 48%) come from the area of medicine, while seven (7/25, 28%) are from computer science, and the rest (6/25, 24%) are from interdisciplinary work. Journals were the primary type of publication (20/25, 80%), followed by conferences (5/25, 20%). The selected studies are mostly authored by researchers from Europe (12/25, 48%), followed by the United States (11/25, 44%).

### 3.1. RQ1: Which Types of Technologies Have Been Developed to Support the Health Management of Older Adults with Multiple Health Conditions?

The types of interactive health technologies mentioned in the literature may be grouped into three categories (see Table 2). First, we have technologies that are based on applications and websites. Some of these technologies have focused on formative and organizational support [31,32], facilitating attendance at appointments [33], integration of care and coordination between health professionals and older adults [34,35,36,37,38], or medication management [39,40,41]. Smartphones, tablets, and computers have been used to implement these systems. This group, which is the most frequently mentioned, focuses on displaying and managing health information. The second group corresponds to wearable sensors; the technology focuses on measuring or detecting information that relates to the physical world, e.g., falls [42], foot movement [43], and biometric parameters [44], as well as teaching behaviors and integrating care into the home [45]. The last group includes devices composed of hand-held and digital parts. These devices focus on cognitive and sensory assistance [41].

In terms of how technology is tailored to support multimorbidity, we found that 8/25 (32.0%) papers presented technologies primarily oriented to the management of a single disease, but were evaluated in a context of older people with multimorbidity. The remaining research (17/25, 68.0%) developed or improved technology to support the management of multiple diseases in older people. These technologies are described in Table 3. For example, a health management system incorporated a social network component called Clinical Wall and the Clinical Decision Support (CDS) system to care for adults with multimorbidity [35], and a medication management application implemented medication information retrieval, doctor visit preparation, and information on when to seek assistance to support adults in reducing medication errors [39].

We classified multimorbidity support in five types of areas in which the technology helped or guided older adults (see Table 2). Of the 17 articles with specific support for older adults with multimorbidity, two (2/17, 11.8%) focused on medical records [42,44], six (6/17, 35.3%) on facilitating communication with others [34,35,36,37,38], nine (9/17, 53.0%) examined technology as treatment guides [31,32,33,34,36,38,47,48,50], one (1/17, 5.9%) on providing access to health information (information access) [35] and three (3/17, 17.6%) on guiding medication management [39,40,41]. Two of the articles provided two types of support (communication and information access [35], and communication and treatment [38]). It is important to note that the provided support is not only related to the older adults, as some of the studies also focused on their caregivers (e.g., [38,55]).

Regarding the relationship between the type of technology and the support for multimorbidity, the researchers choose technologies that are appropriate for the intended multimorbidity support. For example, all the communication technologies, i.e., those that aim to help older adults to communicate and obtain information about their health (e.g., videoconferencing applications), are implemented through mobile applications (3/5, 60%) or websites (2/5, 40%), and all of those that aim to update medical records and health data are based on wearable sensors (2/2, 100%).

### 3.2. RQ2: What Are the Expected Health-Related Outcomes of Technological Interventions for Older Adults with Multimorbidity?

We wanted to know which health improvements or outcomes are the goals of the interventions described in the selected papers. The studies focused primarily on five dimensions of health: (1) physical, (2) mental, (3) social, (4) emotional, and (5) environmental (see Table 4). The largest number of studies referred to improved social interaction (11/25, 44.0%) and mental well-being (11/25, 44.0%); the first aspect refers to communication with family, friends, or medical staff. Mental well-being is especially important for older adults because it can affect health and quality of life. Following is the aim of improving physical aspects, i.e., functional improvements, with nine articles (9/25, 36.0%). Finally, some studies focused on emotional (3/25, 12.0%) and environmental well-being (1/25, 4.0%). Some studies focused on improving more than one health dimension [31,36,39,44,45,48,51,52,53].

Although we aimed to study the effects of the technological interventions on health, the selected papers did not measure this as an outcome, rather focusing on which aspect of health is supported by technology. Therefore, it was not possible to accurately describe health-related effects of the interventions.

### 3.3. RQ3: How Are Technologies for Older Adults with Multiple Health Conditions Evaluated?

Regarding the assessment methodology used to evaluate the proposed technologies, this review considered three main aspects for the analysis: the methodological approach, the sample size, and the age of the participants (see Table 5).

The observed studies were almost evenly distributed between qualitative (9/25, 36.0%) and quantitative methods (10/25, 40.0%). Three studies used mixed methods (3/25, 12.0%), and three studies (3/25, 12.0%) did not carry out an evaluation, as they were system designs or research proposals.

Regarding study design, only one study was part of a randomized control trial (RCT) [41], while another was initially designed as such but randomization failed and the study was considered to be a prospective cohort study [52]. One study was a pre/post pilot study [32]. Several papers present interview or focus groups studies [33,34,36,37,40,45,50,53], with some also including co-design workshops or participatory design [39,49,51], with the goal of understanding some experience—e.g., how older adults perceive care and case management [34], how care navigation is experienced [33], the self-management of medication [40]—or with the goal of understanding usability and interface preferences, e.g., [36]. Other studies share the latter aim but employ surveys and questionnaires, e.g., the Technology Acceptance Model (TAM) [35], as well as others [43,46,48,54,55]. The final two studies used data from system use or testing to understand system effectiveness [38,42]. Therefore, most studies are not experiments nor quasi experiments, but rather evaluations of technology or explorations of user needs or experiences.

Almost all of the studies that had participants used non-probability sampling (18/22, 81.8%). The only exceptions were the RCT [41], the prospective cohort study which used a modified randomization due to a breach in its implementation [52], and one study which used stratified probability sampling [36]. One study did not provide enough information about the sampling method, only specifying that it was a complex sampling design [48]. The composition of the sample of participants is directly related to the focus of each study, i.e., some only include older adults as the final user and others also include e.g., caregivers or healthcare professionals. Four of the studies [35,38,42,51] address the development of technology for older adults, but they do not explicitly state the age of the participants.

The number of participants varied between one small study with less than 10 participants 0–9 (1/25, 4.0%), to medium-sized studies with 10–49 participants (11/25, 44.0%), and larger studies with 50–99 (2/25, 8.0%), and over 100 (8/25, 32.0%) participants. There was no clear correlation between study size and methodology. The averages of population age in the reviewed articles are the following: 60–69 years (4/25, 16%), 70–79 years (9/25, 36%), and 80–90 years (2/25, 8.0%). However, some did include participants who were not older adults, e.g., in one study the inclusion criteria was vascular surgery patients over 18, even though the average age of participants was over 70, and the youngest participant was 41.

## 4. Discussion

### 4.1. Literature Review Overview

The aim of this work was to study which interactive health technologies for older adults with multimorbidity have been proposed, how their outcomes are measured and how they are evaluated. With this aim, we conducted a systematic literature review of four computer science and medical databases, obtaining 681 papers, out of which 25 studies were analyzed in this review. We found evidence of the use of a variety of technologies; spanning from applications and websites, to wearable sensors and devices. These interventions have been used to support multimorbidity through facilitating communication, updating medical records, providing treatment guides and access to health information, and enabling medication management.

The reviewed studies aimed at supporting the physical, mental, emotional, social and environmental health of their users. This holistic focus that includes mental health is relevant due to the link between the mental health of older adults and mortality [56], and also because previous studies that have shown that people with multimorbidity are more likely to suffer from depression and anxiety, which can further exacerbate their physical conditions [57].

Taking into consideration the aging of society in general, and the accompanying cultural transformation, there should be an increasing trend of new technologies being designed, adapted, and oriented mainly for older adults [18]. During the last two years of reviewed papers—2018 and 2019—there seems to be a slightly increasing trend in research regarding technologies for multimorbidity (while in 2020 the COVID-19 pandemic began and research interests may have shifted). An upward trend in research on other technologies for older adults—e.g., home health monitoring systems—has been noted [58].

There are multiple challenges to multimorbidity that make self-management difficult for patients, e.g., the compound effects of conditions and medications, the burden of medications, and difficulties in communicating with healthcare providers, among others [59]. However, most healthcare processes still treat patients as though they had a single disease and ignore these complex interactions [3]. Our study found evidence of this, as 8/25 of the interventions were created for a single disease, even if they were evaluated in a context of multimorbidity.

Recent studies have proposed self-management guidelines for patients e.g., establishing disease and treatment burden and the support patients need to manage their conditions, establishing priorities of importance, setting up medication support and care plans, among others [60]. We wanted to find evidence of how interactive health technologies could specifically focus on the challenges of multimorbidity; and we found efforts tending to integrate information for older adults (see Table 3), which is a step in the right direction when dealing with the complex, fragmented information from several chronic conditions.

### 4.2. Limitations

Some limitations should be taken into account when evaluating the results of this work. We include only papers written in the English language that have been published between 2009 and 2019. Only ACM digital library, IEEExplore, Science direct, and Pubmed sources have been searched; other relevant material may exist in other databases, e.g., PsycINFO. Furthermore, our choice of keywords may have restricted our findings—e.g., the word “technology” was used to represent all types of interactive health technologies, while previous research may have used words such as “digital solutions”, “mhealth”, or directly named a technology.

As a limitation of our study, it should also be taken into account that most of the included studies come from the United States and Europe (consequently, the participants are from those countries). When designing systems, cultural aspects need to be taken into consideration, and in this regard research found in this study is overwhelmingly from the Global North; while the reality of access to and adoption of technology in the Global South may not be represented through these results. Therefore, generalization to other populations should be made with caution and more research is needed to account for and design health technology to support the needs of older adults with multimorbidity in the Global South.

Finally, we did not assess the quality of the selected studies and did not compare the effectiveness of the technologies, as not enough data was available for this purpose. This makes it difficult to provide solid recommendations regarding the best technologies for older adults with multimorbidity. It is important for future studies to provide clear data on the effectiveness of the proposed interventions. In this regard, we found only one RCT within our selected papers. To truly understand the effectiveness of proposed interventions, further experimental work is needed.

### 4.3. Requirements and Challenges for Interactive Health Technologies for Multimorbidity

From this work, we derive four requirements, or challenges, that are present in research on interactive technologies for older adults with multimorbidity: increasing integration, improving ubiquity, personalization, and older adult-centered design. We believe these are relevant as insights that should be taken into account by future researchers. Each is described below.

#### 4.3.1. Increasing Integration

Some of the challenges of multimorbidity stem from managing several illnesses, each with their medication, healthcare team, and related information. The proposed systems in this review usually aim for one part of the integration—e.g., they integrate medication management for several illnesses, or they provide communication with the healthcare team, or they gather data to update medical records, but they do not cover all types of support simultaneously (see Table 2). An integrated system that centralizes all information, and ideally also integrates with healthcare providers’ systems, can provide patients with a rich overview of their health as well as better support their needs.

#### 4.3.2. Improving Ubiquity

The technologies found in the reviewed papers range from mobile and web applications to tangible devices and wearable sensors. Although these solutions cover a range of technologies, special consideration should be given to making the management of multimorbidity an ubiquitous and pervasive technology, i.e., made to disappear into everyday routines effortlessly. This does not necessarily mean making technologies invisible, which could make users forget to use them or hinder adoption, but rather, incorporating them into existing practices so they are naturally part of users’ lives. Examples of such interventions are fall detection systems, or smart medication boxes; however, multimorbidity can add extra challenges (e.g., in detecting medicine interactions) and careful consideration should be given to this aspect when designing interactive technologies for these users.

#### 4.3.3. Personalization

Often, patients are treated by healthcare providers as though they had a single disease, only focusing on that provider’s specific discipline. However, multimorbidity means that patients may have unique combinations of chronic illnesses and therefore unique—and often conflicting [61]—needs. Some existing approaches aim to provide personalized care (e.g., [31]), which is especially relevant in a multimorbidity setting.

#### 4.3.4. Older Adult-Centered Design

Previous research has found that many older adults have positive attitudes towards technology and high levels of digital skills [62], but the barriers of adoption may present a challenge to some of them [45]. Older adults are a heterogeneous population with differing technological skills and interests; e.g., older internet users differ in their knowledge of the web, and skills vary both within and between age groups [63]. In order to design and implement health technologies for older adults, especially adults with multimorbidity, it is necessary to look beyond age range considerations and take into account individual differences in technological literacy, cognitive abilities, and physical limitations.

## 5. Conclusions

The number of older adults will continue to increase in the coming years, as will multiple chronic diseases. The design, implementation, and evaluation of technological tools aimed at older adults with multiple chronic diseases is required to enable this population to remain independent and maintain a good quality of life. Although slightly increasing in recent years, the development of technologies for older patients with multimorbidity remains low. We present a discussion of how technologies for multimorbidity should be designed in the future, to be personalized, ubiquitous, older-adult-centered, and integrated. This review highlights the importance of generating technologies that are directly focused on older adults’ needs.

## Figures and Tables

**Table 1:** Search keywords aligned to PICOC.

Criterion	Description	Keywords
Population	Older adults	Older, senior, elderly
Intervention	Health Management Technologies	Technology
Comparison	—	—
Outcomes	Effects on health	—
Context	Multiple health conditions	Multimorbidity, multiple chronic conditions, multiple diseases, comorbidity

**Table 2:** Technologies to support health management of older adults with multiple chronic conditions (Y = yes).

Paper ID	Type of Technology				Multimorbidity Support				
	Mobile Application	Website	Wearable Sensors	Device	Medical Records	Communication	Treatment	Information Access	Medication Management
[42]			Y		Y				
[33]	Y	Y					Y		
[34]	Y					Y			
[46]		Y					Y		
[45]			Y						
[35]		Y				Y		Y	
[39]	Y								Y
[40]	Y								Y
[36]		Y				Y	Y		
[37]	Y					Y			
[41]				Y					Y
[31]	Y						Y		
[32]	Y						Y		
[47]	Y						Y		
[48]	Y	Y					Y		
[38]	Y					Y	Y		
[49]	Y								
[50]		Y					Y		
[44]			Y		Y				
[51]						Y			
[52]	Y	Y							
[53]									
[54]	Y								
[43]			Y						
[55]	Y								

**Table 3:** Technology tailored to multimorbidity (17 studies).

Study	How Does Technology Support Multimorbidity?
[33]	Healthcare and social care systems integrated navigation and communication support
[34]	Care and case management (CCM) using video conferencing
[35]	The care system integrated the social network component, called the Clinical Wall, and the Clinical Decision Support system.
[39]	Medication management application provides medication information retrieval, doctor visit preparation, and information on when to seek assistance.
[40]	System to provide links to medication information, facilitate communication between patients and physicians and pharmacists, and facility to selectively disclose medication information to different physicians.
[36]	Health portal that integrates e-mail, pharmacy and lab results.
[41]	Drug-handling device includes cognitive and sensory assistance
[31]	Personalized health care integrates appropriate personalized health technologies, standards of care and health planning
[32]	System with integrated medical and psychiatric self-management intervention.
[47]	Improved diagnosis by transferring clinical information (allergic rhinitis and asthma) from MACVIA-France’s EIP on AHA (Allergy Diary) to other sites.
[38]	System with care plan and a communication channel (in the registries the professionals interacted on the patients’ data).
[49]	System integrates goal setting, combined with progress feedback.
[44]	A television that records biometric parameters and displays health-related alerts and recommendations and environmental sensor logs.
[52]	Structured telephone support, with reminders and follow-up that allows remote self-management and transmission of clinical information.
[54]	System with medication reminders, pill dispensing assistance, medication log, medication position and forgotten medication alerts.
[43]	Fall arrest system included a light path along with the telecare service (the telecare service consisted of a remote intercom, an electronic bracelet).
[55]	System that allows taking photos, asking health-related questions and coordinating with caregivers.

**Table 4:** Considered aspects of older adults’ health when using technology for multimorbidity support.

Health Dimensions	Studies	Count
Social	[31,33,34,35,36,38,39,45,46,50,51],	11
Mental	[32,37,39,40,41,45,48,49,52,53,54]	11
Physical	[42,43,44,47,48,49,52,53,55]	9
Emotional	[31,36,51]	3
Environmental	[44]	1

**Table 5:** Methods used to evaluate interventions. Articles [31,44,47] are proposals and therefore were not evaluated.

Study	Methodology			Sample Size				Average Participant Age		
	Quantitative	Qualitative	Mixed	0–9	10–49	50–99	>100	60–69	70–79	80–90
[42]	Y				Y					
[33]			Y			Y				
[34]		Y			Y				Y	
[46]			Y				Y			
[45]		Y					Y		Y	
[35]	Y									
[39]		Y			Y				Y	
[40]		Y			Y					Y
[36]		Y			Y				Y	
[37]		Y			Y				Y	
[41]	Y						Y			
[32]	Y				Y			Y		
[48]	Y						Y			
[38]	Y									
[49]		Y			Y				Y	
[50]			Y	Y					Y	
[51]		Y			Y					
[52]	Y						Y		Y	
[53]		Y			Y			Y		
[54]	Y				Y			Y		
[43]	Y						Y			Y
[55]	Y					Y		Y		

## Data Availability

Data sharing not applicable.

## Abbreviations

The following abbreviations are used in this manuscript:

SLR	Systematic Literature Review
RQ	Research Question
PRISMA	Preferred Reporting Items for Systematic Reviews and Meta-Analyses
PICOC	Population, Intervention, Comparisons, Results, Context
CDS	Clinical Decision Support
